# Nurses’ perception of epidural services in a Johannesburg academic setting

**DOI:** 10.4102/curationis.v47i1.2534

**Published:** 2024-08-07

**Authors:** Nomalungelo C. Mbokazi, Janine Wagner

**Affiliations:** 1 Department of Anaesthesiology, Faculty of Health Sciences, University of the Witwatersrand, Johannesburg, South Africa

**Keywords:** labour epidurals, education and training, service delivery, nurses, academic hospital

## Abstract

**Background:**

Labour pain is associated with detrimental maternal and foetal physical and psychological effects. Labour analgesia is a basic right for all women and labour epidural analgesia has been accepted as the gold standard for providing such, with reported improvement in patient satisfaction. In South Africa, studies have shown that labour epidural rates are low. At an academic hospital in Johannesburg, a 24-h labour epidural service combined with an awareness campaign and educational programme (LEAP) was initiated with the aim of improving labour epidural rates. Results showed a short-lived uptake with a subsequent decline.

**Objectives:**

This study explored the experiences of labour ward nursing staff regarding the labour epidural service at this academic hospital including perceived limitations and possible recommendations regarding improving service provision.

**Method:**

A qualitative, descriptive and exploratory study was conducted. Purposive sampling was used with semistructured, audio-recorded individual interviews, thematic analysis was performed using Braun and Clarke’s six-phase approach.

**Results:**

The key theme is required education and supervision of epidural insertion (see page 3), management of childbirth and challenges related to epidural service provision.

**Conclusion:**

A positive sentiment was expressed by the participants; however, deficiencies in the service such as shortages of experienced personnel, work constraints and insufficient training may be affecting service sustainability. Further studies are recommended to form guidance towards the development and implementation of interventions to improve service delivery.

**Contribution:**

Provision of continual training and increased staffing of healthcare personnel will help improve the sustainability of the labour epidural service.

## Introduction

Labour is a painful emotional experience, which may adversely affect the mother and the foetus acutely in the long term (Labor & Maguire 2008:15). It has been shown that most women experience moderate to severe pain during labour, but labour has been described by some women as the most painful experience of their life (Kroh & Lim 2021:45).

There are various non-pharmacological and pharmacological methods of labour analgesia (Jones 2012:101). However, labour epidurals are regarded as the gold standard for the provision of analgesia during labour (Sng & Sia 2017:15). Labour epidurals have been reported to improve patient satisfaction, prevent post-partum depression and are associated with decreased levels of paternal or partner anxiety (Capogna, Camorcia & Stirparo 2007:110; Ding et al. 2014:383).

Labour epidural analgesia is widely available in high-income countries; however, in low- and middle-income countries, labour epidural rates have been generally reported as low (Fan, Gao & Yang 2007:205; Hu et al. 2014:2181; Traynor et al. 2016:1939). In South Africa, studies conducted in the Western Cape province by Van Zyl et al. (2017:156) and in the Gauteng province by Leonard et al. (2018:52) and Wagner et al. (2021:99), have shown that labour epidural rates are low in both the public and private sectors. Although the cause of low labour epidural rates may be multifactorial, the initiation of a 24-h labour epidural service combined with an awareness campaign and educational programme (LEAP) conducted at an academic hospital in Johannesburg, Gauteng, resulted in a significant increase in the uptake and acceptance of labour epidurals. However, the improved labour epidural rate post-LEAP was unsustainable (Wagner 2021:505). Multiple factors, including nursing staff’s attitudes and perceptions, may be affecting service uptake. An in-depth understanding of the labour ward nursing staff’s perception of the epidural service is needed so as to gain insight into possible strategies that could be employed to improve the sustainability of services.

The aim of this article was to describe the experiences and perceptions of labour ward nursing staff regarding the labour epidural service at this academic hospital, including perceived limitations of the service and possible recommendations with respect to improving service provision.

## Research methods and design

### Research design

The study utilised qualitative, descriptive, exploratory research methodologies. Thematic analysis, using the generalised inductive approach and Braun and Clarke’s six-phase approach, was utilised (Braun & Clarke 2006:77).

### Study setting

This academic hospital located in Johannesburg is a regional level hospital situated in the Gauteng province that primarily caters for the obstetrics, gynaecology and paediatric specialities. It is affiliated with the Faculty of Health Sciences at the University of Witwatersrand (Wits) and currently offers a 24-h labour epidural service.

### Population and sampling

A purposive sampling technique was used in the study. Purposive sampling is a research technique that is used in qualitative research to intentionally select individuals and sites to learn and understand a central phenomenon (Creswell 2012:206). This sampling method was used as it was most aligned with the research study at hand. The participants who were chosen for the study were qualified midwives and professional nurses working in labour ward who were employed on a permanent basis by the hospital. These nurses were chosen by the researcher as they were considered to be the individuals who would potentially offer the most in-depth information regarding the epidural service as they were intricately involved with the service and would offer their first-hand experience and perception of the epidural service.

The first author identified that there were 19 permanently employed qualified midwives and professional nurses working in the labour ward, excluding the ward operating manager. An invitation to participate was sent to all 19 participants.

### Data collection

One-on-one interviews were conducted by the researcher in the labour ward nursing tea room. This venue was chosen as it was familiar to the participants. Refreshments were served during the interviews. The data were collected by the first author, who is a trainee anaesthetist in the Wits Department of Anaesthesia.

The interviews were in the format of one-on-one audio recordings, with the questions presented in a semi-structured in-depth format (see Appendix 1). The overarching question that was asked was, ‘What can you tell me about your experience with the labour epidural service in your labour ward at this academic hospital?’ The semi-structured interview format was designed to stimulate conversation and allowed the conversation path to develop organically as led by the participants. Although the interviewer was proficient in English, IsiZulu and IsiXhosa (some of the predominant languages in Johannesburg), interviews were conducted in English. In addition to the audio recording, there were field notes recorded by the first author. The audio-recorded interviews lasted an average of 10 min (range of four to 23 min). Audio-recorded interviews were then transcribed verbatim by the first author.

All 19 participants were interviewed as it was deemed prudent to get all the participants’ individual experiences. Having interviewed all 19 participants, data saturation was reached as there were no new emerging themes (Corbin & Strauss 2008:65; Saunders et al. 2018:1893).

Only qualified midwives and professional nurses who were permanent employees in the labour ward unit were included in this study. By an iterative process, the first author found that there were no new emerging themes after having interviewed 15 participants. A decision was taken to continue with the rest of the interviews to ensure validity.

### Data analysis

Audio recordings of all the interviews were transcribed by the first author verbatim. Multiple sessions of audio listening were conducted to ensure the accuracy of transcription. The data were then coded, codes were then grouped to categories and finally themes and sub-themes. Coding of themes and sub-themes was reviewed with the second author to ensure congruency. A general-purpose thematic analysis method was used, and during the coding stage, an inductive method of code generation was used. The analysis process was guided by Braun and Clarke’s six-phase approach (Braun & Clarke 2006:77). This entailed the first author familiarising herself with the data during the interview process, throughout the processes of audio recording, transcription and through the process of code and theme generation. Thereafter, the initial codes were generated from the transcript. Through an iterative process, the initial coding led to the formation of themes, review of the themes that had emerged resulted in the formation of thematic maps and further refinement of the themes resulted in the formation of sub-themes. Finally, all the phases were collated to form a final analysis.

To ensure qualitative research trustworthiness, Lincoln and Guba’s (1985:289) evaluative criteria was used:

- Credibility was ensured by peer debriefing. This was performed by having two sessions with colleagues, where an oral summary of the research findings was shared by presenting emerging themes.
- Confirmability was performed by an audit trail and reflexivity. An audit trail was carried out by having field notes and raw data in the form of audio-recorded interviews, which were transcribed verbatim, followed by data reduction and analysis in the form of coding and theme formation. Reflexivity was performed by acknowledging that the first author was a training anaesthetist in this research institution. This brought about the biases that the author may have about the working environment and the participants. The author acknowledges that there may be a level of unconscious bias formed from the previous experiences and assumptions but endeavoured to keep an objective view during the data collection process. This included establishing rapport with participants and creating a comfortable environment for the discussions.

### Ethical considerations

An application for full ethical approval was made to the Human Research Ethics Committee (Medical) and ethics consent was received on 04 October 2022. The ethics approval number is M220758. Also, written consent for the study was obtained from the research committee at the academic hospital in Johannesburg, the Anaesthesia Head of Department and the Operating Manager in charge of the labour ward.

The study was conducted using the South African good clinical practice guidelines (Department of Health 2020:6) ensuring that participants fully understood what the study entailed. This was provided in the form of an information sheet, which explained the purpose of the study; and included both authors’, N.C.M. and J.W. details.

Participants’ privacy and anonymity were respected by deidentification of personal information from the recordings and transcripts. Further protection of the participants’ identity was ensured by storing the transcripts and recordings in a password-protected folder that can only be accessed by the authors of this article.

## Results

### Participants’ demographics

The socio-demographic characteristics of the participants are summarised in Table 1.

**TABLE 1:** Participants’ socio-demographic characteristics.

Participant number	Age range in years	Gender	Qualification	Years of experience	Interview time
1	36–40	Female	4-year diploma in nursing	6–10	11:40
2	36–40	Female	4-year diploma in nursing	11–15	23:27
3	61–65	Female	BSc Nursing	> 20	06:25
4	26–30	Female	4-year diploma in nursing	1–5	18:13
5	41–45	Female	4-year diploma in nursing	11–15	12:17
6	31–35	Male	4-year diploma in nursing	1–5	08:28
7	51–55	Female	1 year diploma in midwifery (post-graduate diploma)	> 20	12:15
8	36–40	Female	4-year diploma in nursing	6–10	04:47
9	20–25	Female	4-year diploma in nursing	1–5	06:47
10	26–30	Female	4-year diploma in nursing	1–5	05:57
11	41–45	Female	BSc Nursing	11–15	21:08
12	31–35	Female	4-year diploma in nursing	6–10	10:02
13	36–40	Female	1 year diploma in midwifery (post-graduate diploma)	6–10	10:46
14	36–40	Female	1 year diploma in midwifery (post-graduate diploma)	11–15	10:18
15	20–25	Female	4-year diploma in nursing	1–5	07:57
16	26–30	Female	BSc Nursing	1–5	05:56
17	26–30	Female	BSc Nursing	1–5	07:21
18	41–45	Female	4-year diploma in nursing	6–10	10:41
19	26–30	Female	4-year diploma in nursing	1–5	04:51

As the interviews progressed, a few emerging themes arose. These themes were then reflected upon by the first author, using an iterative process. The following themes were found to be predominant: required education and supervision of epidural insertion, management of childbirth and challenges related to epidural service provision. The outline of the themes and sub-themes is summarised in Table 2.

**TABLE 2:** An overview of the themes and sub-themes.

Themes	Sub-themes
Required education and supervision of epidural insertion	Education and training of anaesthetic doctors Education and training of the nurses Patient-centred education
Management of childbirth	The labour process The effects of epidurals on the labouring woman and baby Management of patients
Challenges related to epidural service provision	Language and culture of patients Ethical considerations regarding patient consent Work environment Service availability

### Required education and supervision of epidural insertion

During the interviews, participants expressed the importance of training of all healthcare personnel, including the doctors regarding epidurals. There was also identification that the educational component of the epidural service and overall knowledge was an evolving phenomenon that required continual education. From this, the following sub-themes emerged: education and training of anaesthetic doctors, education and training of the nurses and patient-centred education.

#### Education and training of anaesthetic doctors

It was noticed that the institution that is currently providing the epidural service is an academic one, and that current supervision during training may be insufficient. Furthermore, anaesthetists’ epidural insertion skills varied and this affected their performance and overall confidence. There was a sense that if the doctors did not have any prior experience or had minimal experience with insertion of epidurals, the presence of a senior doctor was needed to help assist the training doctor:

‘For me sometimes I like feel with certain doctors like when it’s their first rotation and like they have to do their first epidural on their own, I feel like it would be nice if someone would just accompany them because you can see that they are scared.’ (P17)

Some of the participants expressed that senior supervision and oversight would help improve the overall provision of epidurals to patients. This was seen as an intervention to improve both the learning of the anaesthetic doctors and the nurses themselves. Of note was that the patients were sometimes being deprived of the service because junior anaesthetists were not able to obtain senior support and assistance:

‘We have had cases where the epidural didn’t go in and then now the patient has to suffer because the anaesthetist has failed. So the second opinion, it’s like with me if I do a PV [*per vaginal examination*] and then I feel that I can’t find the readings that I need to find. I go call for a second opinion on the more experienced person.’ (P18)

#### Education and training of the nurses

Participants discussed an array of topics regarding their training, which included their undergraduate, current and continuing education. The participants expressed that they did not have thorough undergraduate training regarding epidural monitoring. It was found that most of the hospitals that they trained in did not have epidural services and so as a result they were taught about the procedure theoretically without having any practical exposure:

‘Honestly speaking I trained in Limpopo so eish there aren’t lot of resources in Limpopo, so I didn’t know anything about epidurals maybe they just taught us, but I had never seen any epidural before.’ (P6)

Most participants only had exposure to the epidural service once they started working at this academic hospital or in the private sector:

‘As midwives we are not trained that much. I learned epidural when I was in private clinic, I did … yah.’ (P3)

The participants found that their current knowledge of epidurals and the service itself had improved with continual exposure working in the labour ward unit that offered the service. Of note was that continual training for nursing personnel was still lacking.

They expressed that their current knowledge was from day-to-day exposure, but there had not been any formal teaching regarding labour epidurals that was directed to the participants caring and nursing the patients:

‘Maybe an in-service training about, more about the epidural so that we can get better understanding of it. Because what I have learnt, I have learnt along the way and not, basically there was never an in-service training saying this is an epidural, this is what it does, and then I have never received such.’ (P16)

The participants who had been working at this academic hospital for a while noticed that there had been previous training and education dedicated to the nurses working in labour ward, which was provided by the doctors as well as the nurses who had an interest and in-depth knowledge about epidurals. This had, however, overtime stopped and at present the participants were currently not receiving any ongoing training and education:

‘We did have a Sister, but she is retired now. She used to work in labour ward, she was an epidural Sister, she didn’t do the epidural, the doctors would, but she knew everything about epidurals she used to give … if you were interested and you wanted to know about epidurals, she would give you lectures and things like that you know.’ (P7)

#### Patient-centred education

Participants expressed that antenatal patient education gave patients a better understanding of what labour epidurals are along with the benefits and the anticipated side effects. Once the patient was in labour, informed consent was usually difficult, especially when a new concept such as an epidural was introduced in the terminal phase of the pregnancy.

Another point raised was that giving antenatal education offered psychological preparation for the patients before labour, especially if given in a language that the patient understood. Participants reported that antenatal patient education was insufficient:

‘I wish it can be included during your ANC (antenatal care) visits. You know when they give them the leaflet of the danger signs and things. I wish they can also include this pamphlet of epidural because there is this nice one that is having all these languages that you can read out what is epidural.’ (P4)

### Management of childbirth

During the interviews, the participants delved into how they managed patients in labour, particularly those who had labour epidurals. The process of managing child birth brought about the following sub-themes: the labour process, the effects of epidurals on the labouring woman and baby and management of patients.

#### The labour process

As the interviews took place, the process of labour was described by the participants in varying aspects. As the topic came about, participants had different points regarding pain relief and the process of delivery. Participants communicated that there were different modalities of pain relief, which in their practice mainly included pethidine, use of labour epidurals, as well as other less commonly used means. Participants indicated that prior to exposure and familiarisation with labour epidurals, their main source of pain management was pethidine. This was in keeping with what they were most commonly exposed to in their training:

‘When I came in here I was totally not exposed and we were using like pethidine and hydroxyzine for pain and that was it.’ (P13)

The participants spoke about the varied effects of pethidine from patient to patient. Participants communicated concerns regarding the effects of pethidine on the mother and the foetus, with the mention of pethidine making some patients very heavily sedated and that at times there was development of foetal compromise after administration of the drug:

‘When we give pethidine and whatever we give, it will only work for an hour or so and then sometimes 30 minutes or so.’ (P7)

With respect to labour epidurals as a modality for pain relief, participants communicated that from their experience, they have seen that it helped relax and calm the birthing mother, which in turn created a better working environment. Additionally, its use was observed to be of benefit in the patients with comorbidities such as hypertension where its use prevented excessively elevated blood pressures during labour. The analgesic provision for procedures such as an episiotomy repair was also highlighted by participants:

‘With an epidural everybody is calm, mom gets time to sleep and relax, so she actually has strength and like the mental ability away from all the stress to say okay, they are telling me to push now even though I can’t feel the pain, okay I am going to push, I am going to push and cooperate.’ (P17)

Some participants did, however, express that sometimes the epidural also failed or did not work. This then manifested itself as a partial or complete block failure, which was in effect not ideal for all involved as the patients continued to experience unmanaged pain:

‘The issues are that sometimes only one side goes numb.’ (P14)

#### The effects of epidurals on the labouring woman and baby

During the interviews, the participants discussed the delivery process, which mainly involved patients who had received labour epidurals. It was found that patients who received epidurals had rapid progression of the first stage of labour. This was attributed to increased cervical dilatation in the presence of the epidural:

‘And also sometimes, some patients after epidural they progress so fast … yah, this is the best.’ (P6)

The participants noticed the rapid progression of the first stage of labour, but this was in contrast to the second stage of labour. Regarding the second stage of labour, participants expressed that there was a loss of the urge to push or bear down, which inevitably led to a prolonged second stage of labour. For some of the participants, this was one of the deterrents for using epidurals. Not all the participants were deterred, with some finding that there were different measures they had utilised when there was a longer second stage of labour than anticipated:

‘Just when the patient is about to push, now you have a problem because she doesn’t have the urge. But if you allow the patient to … give the patient time even though she is fully dilated don’t start by telling her to push, just let her be, when the pain is strong enough, even with the epidural she will push but it takes time.’ (P11)

Participants generally communicated that epidurals do not affect the foetus, this was in part one of the reasons why some of the participants expressed a preference to it. There were, however, some participants who reported having observed poor foetal outcomes, which they had attributed directly to the epidural and the need for assisted deliveries:

‘The main thing that I like uhm … epidural, it doesn’t affect baby in any way.’ (P2)

‘I started working in labour ward January this year and ever since I have never or should I say in ten patients that I have received or nursed who have had an epidural, maybe two delivered successfully, the rest has to be an assisted.’ (P5)

Participants expressed concerns regarding motor blockade and residual effects post-delivery, which were viewed as major challenges for patients. Participants described the need for longer monitoring periods post-delivery as well as delayed discharge:

‘The only challenge that I experience sometimes is overdosing a little bit because some patients after delivery they are unable to walk, so that is one of the challenges I have.’ (P8)

#### Management of patients

When it came to management of patients, participants described the unique environment they are in where they have two patients to manage at any given point. This meant that for every decision that they took, they not only considered the mother, but the foetus as well.

When delving deeper regarding the treatment and management of the mother, participants expressed the importance of the overall positive experience that the mother needed to have during childbirth. There were concerns raised by the participants about the overall competency of the anaesthetists and their role as the doctors performing the epidural procedure in the patients’ experience of childbirth:

‘So I think also the level of skill of the anaesthetist plays a big role uhm in terms of the patient’s experience. Because if you are pricking that patient ten times, then it’s a problem, it’s not nice.’ (P14)

The magnitude of pain experienced by the birthing mothers was well understood by the participants. This was based on their own experiences as nurses and from their own personal birthing experiences:

‘Even from i-patient [*the patient*] ne, because usually ama-patient [*the patients*] when they are in labour especially round about abo [*approximately*] five six centimetres dilated ne because the pain is so unbearable nabo [*they*] can’t cope angithi [*right*] … in all honesty.’ (P1)

It was found by the participants that some patients had concerns and fears regarding receiving the epidural. These fears were based on the potential complications that may arise and the thought of the site of insertion. Participants expressed that pre-procedural patient counselling was insufficient and that there were patients who had certain misconceptions about epidurals, which resulted in refusal of the procedure:

‘It’s just the patients, you find that they were misled, the information was not adequate.’ (P18)

### Challenges related to epidural service provision

Participants continually discussed how the service was at present; this also included the challenges they were facing and how they believed the service could be ultimately improved. Following these discussions, four sub-themes became evident: language and culture of patients, ethical considerations regarding patient consent, work environment and service availability.

#### Language and culture of patients

Participants expressed how management of the birthing mothers poses certain difficulties as there are practical difficulties such as language barriers because of the influx of foreign migrants. Most of the patients from other countries did not know any of the locally spoken languages in South Africa and participants expressed that there were major difficulties obtaining the services of interpreters. This then resulted in communication difficulties for the nurses and the anaesthetic doctors trying to offer the service. There was additional concern that some of the patients could potentially agree without fully understanding the risks and benefits:

‘I think because of where we work, and the major problem we have had is like the language barrier. I don’t know really is there not something we can do if the patient can’t really understand and they don’t get access to the service.’ (P14)

Cultural differences between the participants and the birthing mothers created a unique challenge for all involved as some of the participants did not fully understand certain behaviours displayed by the patients. Some participants conjectured that a lack of education played a role, while others thought that certain beliefs were ingrained in those patients, which were very dissimilar to what the women in South Africa were exposed to:

‘So most of the patients they are scared they have those fears and they have to consult their husbands. Because mostly we deal with patients from outside who are very obedient to their husbands, they can’t do anything without the husband’s consent, yah.’ (P9)

Participants expressed concern regarding their role in the management of two patients and difficulty of achieving adequate foetal well-being monitoring in the presence of an uncooperative birthing mother.

Even though participants were acutely aware that the state of labour caused the birthing mothers to be uncooperative, it was still challenging for them to manage. The participants found that with the patients who had received the epidural, monitoring of the foetus was easier. This then led to a better cardiotocography tracing, which enabled all the healthcare providers involved to make an informed decision regarding further management of both the birthing mother and the foetus:

‘Most of our patients are required to be on CFM [*continuous foetal monitoring*] because they are mostly high-risk patients and we need to see how the foetal heart rate is and how the contractions are.’ (P2)

#### Ethical considerations regarding patients consent

As the participants were the primary healthcare personnel taking care of the labouring patients, they raised concerns regarding different scenarios that presented themselves in the realm of ethics in the healthcare environment.

The participants expressed that there were patients who did not fully understand what the epidural procedure entailed and yet still consented to the procedure.

This then brought about issues regarding informed consent especially timing of consent. Some of the participants expressed that during labour some patients are not fully cognisant of the potential complications:

‘They do explain to the patients about the epidural, but I am not sure if our patients, they actually understand because if you tell them I am going to give you an injection, this injection will make the pain to go away. That’s what they are focusing on, any other thing they don’t even care as long as you are going to take the pain away.’ (P11)

The participants also expressed difficulties with certain patient populations where consent could not be taken because of a language barrier. Participants were also not fully informed regarding obtaining consent from women under 18 years of age:

‘And also, with age, if like teenagers were able to give consent, I feel like it would be much better because their pain threshold is so low.’ (P9)

Some participants also raised the concept of patient autonomy being of paramount importance to them. This was expressed as something that is highly respected as the patient’s wishes should always be respected regardless of any personal opinions they may have:

‘But I feel that it’s not for the nurses to decide, it’s for the patient to decide what she wants.’ (P7)

#### Work environment

The discussions that arose from the participants regarding the work environment were focused on the patients who had received the epidural and the unique challenges and positives that they as the people caring for these patients had experienced.

The participants expressed how in their current unit they were faced with the issue of overcrowding and an ever increasing number of patients seeking healthcare services during labour and delivery. Participants found that patients who had received an epidural tended to have a longer second stage of labour, and that this, in a high turnover environment, created an additional challenge to an already overburdened system:

‘And with us and the influx, we are working on time. But other than that, if we were having enough staff, if we were not having so many patients it would be fine, I mean it would be good. It’s just that we tend to rush our patients when they are fully dilated because we want them to push and finish because I am having another patient.’ (P11)

Participants described what they deemed as a calmer working environment when the birthing mother had an epidural.

This was seen as beneficial as it created an environment where both the mother and the participant could focus on the task at hand without any distractions. This resulted in what participants perceived as a decrease in the workload even though the volume of patients delivering was still unchanged:

‘Mina [*I*] think it’s extremely helpful to the midwife and to the patients themselves, because they become less distressed, you’ve got normal vital signs even the baby’s CTG with monitoring iya imphruva [*it improves*] because of that.’ (P1)

#### Service availability

Participants discussed and contrasted how the epidural service was and how it currently is. They found that it was previously only available from 07:00 to 16:00 during the week as there was a shortage of anaesthetic doctors. This resulted in a number of challenges for the participants as some of the patients could not receive the epidurals and with those who had received epidurals were not monitored after 16:00. This also caused some hesitancy with the participants as they did not like the fact that some patients had epidurals removed mid-labour as there were no anaesthetic doctors to monitor the patient.

It was communicated by the participants that currently the service has improved as the doctors are available around the clock during the week and even at night to offer the service to the patients:

‘First time that it was introduced it was a bit hectic to explain to the patient because now mostly you find that they were done from 07:00 to 16:00 so currently of lately it’s nice because you guys are leaving at 19:00 and then at night there is also someone to relieve you.’ (P13)

Participants expressed issues with ethical principles, especially distributive justice. Even with these strides made participants still felt that the service needed to be available on all days of the week as the need for labour epidurals arose at any time of the day, that is to say birthing mothers delivered all the time. Some participants expressed that it was difficult for them to witness some of the birthing mothers experience severe labour pains over the weekends and holidays when labour epidurals were available for their counterparts who had come during the week:

‘I just feel like there should be more availability. Like if there’s more anaesthetists 24/7. I feel like it’s a little bit unfair that I will come today and get the epidural and somebody else comes on Saturday and Sunday and then they don’t get that service. That’s the only thing that I feel is a little bit unfair.’ (P14)

Participants also expressed that service availability was also hampered by shortage of healthcare personnel both from the anaesthetic doctors as well as the nurses. It was continually observed that the patient burden is higher than the available staff to cater for them. Additionally, there were occasional equipment issues in the form of shortage.

It was further observed by the participants that this was sporadic, but when present resulted in the service not being available to the patients:

‘However, the challenge is just shortage of staff more than anything, but the service is quite good where we are trying our utmost best to be excellent.’ (P12)

## Discussion

This study provided an understanding of the labour ward nursing staff’s experience and perceptions regarding the epidural service at an academic hospital in Johannesburg. This is the only public hospital in Gauteng that aims to offer a 24-h labour epidural service. This study was warranted as it was found that the LEAP intervention implemented at this hospital provided only a short-lived unsustainable uptake in the epidural service (Wagner 2021:505). Furthermore, this study sought to gain insights regarding the perceived limitations of the service and possible recommendations regarding improving service provision.

One of the themes that arose was a lack of training and continual education of all healthcare personnel who were intricately involved in the labour epidural service provision.

Continual education and training have been shown to play a pivotal role towards service provision in the units that offer labour epidural services, with the lack of trained personnel resulting in limited provision (Maeda et al. 2019:631; Van Zyl & Burke 2017:156). Participants who had been working in the unit for a long time found that training previously available had stopped over time. This finding underpins an area of potential service improvement. Su et al. (2023:2181) noted that with training, midwives are able to provide an important role regarding supervision and maintenance of epidural analgesia.

Participants conveyed that the training of the anaesthetic doctors was somewhat lacking and more senior oversight was warranted. This was revealed by instances where they had seen and interacted with anaesthetic doctors who seemed to either have minimal or no experience at all when it came to the skill of labour epidural insertion. A study in England showed that training anaesthetic doctors needed to perform at least 10 supervised obstetric epidurals prior to them working alone (Drake, Coghill & Sneyd 2015:951). In another study, epidural insertion proved to be the most difficult skill among trainees (Naik, Devito & Halpern 2003:694); this reinforces the sentiment communicated by the participants that senior supervision is needed as there is no direct oversight by senior doctors from the participants vantage point. An evaluation of and change in the current level of supervision is recommended to ensure good patient outcomes and adequate training of healthcare professionals.

The lack of education and training was also found to be present in the patient population as participants also expressed that there was also a lack of patient education in the antenatal period, which contributed negatively towards service delivery. This negative contribution was found to be in keeping with the research data where a lack of patient awareness in the antenatal period led to a reduced uptake of the available epidural services (Ezeonu et al. 2017:905; Kamakshi et al. 2018:501). The lack of patient education in the antenatal period as stated contributed negatively, resulting in a decreased uptake of the service in the unit as there was a lack of patient awareness and informed consent was difficult to attain in some patients. One study found that during labour, women who were already experiencing labour pain and were then seeking pharmacological interventions potentially did not have prior consideration of these pharmacological options (Sanders & Lamb 2014:1893). It could be postulated that those patients who seemed to lack insight to what would be deemed as informed consent by the participants fell into this spectrum. An evaluation of patient knowledge and current antenatal education is recommended to determine whether amendments are required to ensure adequate patient knowledge and ensure informed consent.

Regarding informed consent, participants found that they played a key role as the immediate healthcare providers who ensured that patients took part and understood processes that were taking place during labour and delivery. This was found to be a critical area by the participants as there were different and unequal power dynamics between the doctors and the patients as perceived by the participants. A study performed in the Eastern Cape showed that a majority of midwives felt that it was appropriate that midwives should seek informed consent for the mode of delivery chosen by the patients (Muthige, James & Morton 2019:229). Current departmental policies and practises should be reviewed, and staff education should be put into place to ensure that consent regarding epidurals is adequately informed.

The participants in this study found that different cultural backgrounds, and in some instances, language barriers resulted in decreased provision of the labour epidural service.

Different factors could be at play, but a study carried out by Kamakshi et al. (2018:501) noticed that different sociocultural backgrounds were one of the main reasons why distribution of information was not through formal antenatal class platforms but through relatives and informal talk. The same factors could have potentially played the same role in this study setting. Departmental policies regarding translation services and the availability of translators should be reviewed to minimise the effect of potential language barriers.

Most participants indicated that their exposure and experience of the epidural service were solely from their current exposure in this academic hospital as they had not had previous exposure during their undergraduate and midwifery training. This was in contrast to the developed world set up where a study in the United States of America found that most of the experienced labour and delivery nurses had prior exposure to epidurals before their midwifery training (Walker, Lannen & Rossie 2014:4). Furthermore, the participants conveyed that there was a general sense of discouragement of labour epidurals by the nursing community. This sentiment was also shown by Aune and colleagues who observed that midwives tend to have their own beliefs and values regarding pain management of labour (Aune et al. 2021:384). It could be postulated that the same sentiment may be shared by the nursing community in this study. Overall, the participants communicated a general positive experience of the current epidural service; this contrasted with the views the participants had previously encountered outside of this unit in the nursing community. Evaluation of the current undergraduate nursing curriculum, inter-disciplinary collaboration and the establishment of regular continual education is recommended to address inadequacies in training and experience.

The current 24-h service has been welcomed by the participants. It was noticed that previous unavailability of the 24-h service had resulted in patients not having the opportunity to be offered the service when it was greatly needed. This negatively affected service provision (Wagner 2021:505). Factors that result in the disruption of 24-h services should be assessed and addressed to ensure sustainability of the current service.

Participants revealed that work constraints in the form of overcrowding resulted in the increased workload for all the healthcare providers. This holds true in the South African setting where research performed by Van Zyl et al. (2017:156) found resource constraints as one of the reasons for decreased utilisation of labour epidural analgesia in their academic unit.

Current levels of staffing should be investigated and adequate nurse-to-patient ratios instituted to ensure service sustainability.

### Limitations of the study

The study is limited in that only the experience of nurses working in the labour ward at this academic hospital was examined, and therefore generalisability may not be possible because of the unique set up of the participants and the environment researched. Moreover, this was a study performed in a unit aiming to provide a 24-h labour epidural service, which contrasts with most labour units in the Gauteng province public sector (Wagner 2021:505).

### Recommendations

This study recommends that a continual education programme for nurses and doctors working in the labour epidural unit needs to be employed to improve the knowledge of all those involved towards service provision. Additionally, increasing staff personnel in the caring of patients would assist the nurses towards improving current service delivery.

Increasing patient awareness and education regarding the epidural services in the antenatal setting will ensure that patients utilising the epidural service are acquainted with and understand the services being offered to them. Furthermore, having translation services will ensure that most if not all patients have access to informed consent during childbirth.

Similar studies can be performed to focus on patients, who will offer their perspective.

## Conclusion

This study sought to describe the experience of labour ward nurses working in an academic hospital in Johannesburg and to identify factors that could be limiting service provision. There was a general positive sentiment expressed by the participants towards labour epidurals such as that seen with pain alleviation for the birthing mother; however, participants highlighted that shortages of experienced personnel, insufficient training and exposure of nursing students to labour epidurals, inadequate continual education, inadequate supervision, inadequate patient knowledge and antenatal education and inadequacies with translation services may be affecting the sustainability of the service.

Further studies are recommended to guide the development and implementation of possible interventions to improve service delivery.

## Appendix 1

**Questions to stimulate conversation and discussion:**

- Can you tell me about your experience with the labour epidural service in your labour ward at this academic hospital?
- What role do you play in the service?
- Is there anything that can be done that could help you get more involved in the service?
- What has been your experience of labour epidurals?
- How does the epidural service affect your ability to care for patients?
- Can you tell me about the difficulties you face when trying to offer the service?
- Can you tell me about the training you got about labour epidurals as a student and after qualifying?
- How do you feel about the knowledge you have about epidurals?
- How do you think the epidural service at this academic hospital can be improved, if at all?

Final question: Is there anything else that you would like to discuss or tell me?
